# Laboratory and clinical reliability of conformally coated subretinal implants

**DOI:** 10.1007/s10544-017-0147-6

**Published:** 2017-01-26

**Authors:** Renate Daschner, Udo Greppmaier, Martin Kokelmann, Sandra Rudorf, Ralf Rudorf, Sebastian Schleehauf, Walter G. Wrobel

**Affiliations:** Retina Implant AG, Gerhard-Kindler-Strasse 8, 72770 Reutlingen, Germany

**Keywords:** Electronic implants, Clinical reliability, Artificial vision, Neuroprosthetics, Retinitis pigmentosa

## Abstract

Despite recent developments and new treatments in ophthalmology there is nothing available to cure retinal degenerations like Retinitis Pigmentosa (RP) yet. One of the most advanced approaches to treat people that have gone blind due to RP is to replace the function of the degenerated photoreceptors by a microelectronic neuroprosthetic device. Basically, this subretinal active implant transforms the incoming light into electric pulses to stimulate the remaining cells of the retina. The functional time of such devices is a crucial aspect. In this paper the laboratory and clinical reliability of the two active subretinal implants Alpha IMS and Alpha AMS is presented. Based on clinical data the median operating life of the Alpha AMS is estimated to be 3.3 years with a one-sided lower 75 *%* confidence level of 2.0 years. This data shows a significant improvement of the device lifetime compared to the previous device Alpha IMS which shows a median lifetime of 0.6 years with a lower confidence bound (75 %) of 0.5 years. The results are in good agreement with laboratory data from accelerated aging tests of the implant components, showing an estimated median lifetime for Alpha IMS components of 0.7 years compared to the improved lifetime of Alpha AMS of 4.7 years.

## Introduction

In the hereditary disease Retinitis Pigmentosa (RP) the photoreceptors progressively degenerate, resulting in blindness in adult life, although the rest of the inner retina (bipolar and ganglion cells) and visual pathway downstream of the retina usually stay mostly intact. Although several promising approaches are studied, e.g. gene therapy (Stieger and Lorenz 2010; Hufnagel et al. 2012; Sundaram et al. 2012), electrostimulation (Schatz et al. 2011) or stem cell therapies (Ramsden et al. 2013) there is no therapy available to treat or cure the disease itself yet. One of the most advanced approaches to help blind RP patients is to replace the degenerated photoreceptors by an electronic chip that stimulates the remaining retina cells by charges – a technology that resulted in CE-marked (Stingl et al. 2013; Zrenner et al. 2011; Humayun et al. 2012; www.pixium-vision.com) and FDA approved products (Ho et al. 2015). Different designs of such retinal implants can be distinguished by the position of the charge-transferring electrodes in: a) subretinal (Stingl et al. 2013; Zrenner et al. 2011), b) epiretinal (Humayun et al. 2012; www.pixium-vision.com; Ho et al. 2015) and c) supra-choroidal (Ayton et al. 2014; Ohta et al. 2007). A review about the several research groups and their different approaches is given in Chuang et al. (2014). Retina Implant AG (Reutlingen, Germany) has early on focused on a subretinal chip. Our designs include 1500 to 1600 on-chip photodiodes, each in combination with an amplifying transistor and a charge-transferring electrode. This combination allows an outstanding number of densely packed electrodes and hence the possibility to achieve a high spacial resolution (Stingl et al. 2013).

For all active implants that are in direct contact with body fluids degradation of materials is a critical issue and the electronics need sufficient protection. To achieve a high reliability and durability of active implants, it is generally approved to put the complete implant in a hermetically sealed housing out of metal or ceramics to protect the included electronics, with only the passive components being outside the housing (Ho et al. 2015; Green et al. 2013). For retinal implants this type of construction could be adopted, but would require one cable connection for each stimulating electrode. Therefore a retinal implant consisting of a system with a hermetic housing and a reasonable number of passive stimulation electrodes would require an excessive amount of feedthroughs – an amount, which cannot be integrated inside the eye realistically.

Our two generations of subretinal implants have 1500 and 1600 stimulation electrodes respectively and a corresponding number of photodiodes on chip. As light has to fall on the photodiodes and the chip with its stimulation electrodes needs to be in direct contact with the retinal tissue, a hermetic housing is not an option. Instead only conformal coatings of the chip can be used to protect it from corrosion. Typical coatings that are used in this environment are a combination of several organic and inorganic materials that are biocompatible as well as robust in harsh environments. Such a non-standard system is a novelty for neural prostheses and the effects of active electronic parts in a wet environment are still under investigation. Especially the type of failure mechanisms that occur in such a system has to be investigated. Typical failure mechanisms of active electronics that are in direct contact to saline solution are corrosion and migration of the conductive materials and delamination between layers due to water diffusion. In contrary to a hermetic housing, the process of corrosion of active electronics is a continuous process that is not exactly predictable. Such a statistical process can be described in reliability engineering by probability distributions that depend on the type of failure mode. From this distribution a characteristic lifetime called the *mean time to failure* (MTTF) can be determined. To give a more vivid value, instead of the MTTF we describe all product lifetimes in this paper in terms of the median value, being the time when 50 *%* of the devices failed.

The visual results of the clinical study are already published in Stingl et al. (2015). In this paper we present the first data about the functional lifetime of the subretinal Alpha IMS and Alpha AMS implant. We estimated the system lifetime from laboratory component tests and compared it with the lifetimes of the implant systems in clinical trials.

## Materials and methods

**Fig. 1:**
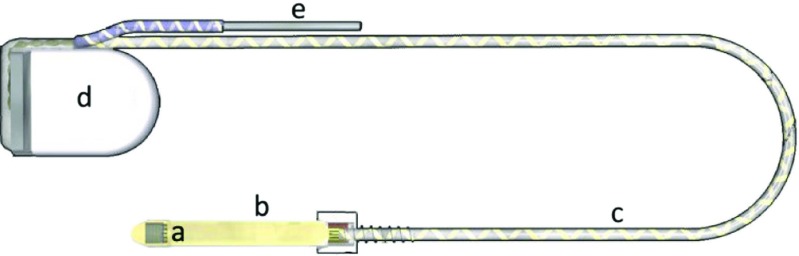
Components of the Alpha IMS and Alpha AMS implant system (**a**: CMOS chip, **b**: foil substrate, **c**: power supply cable, **e**: return electrode, **d**: ceramic housing)

In this publication two product generations are considered, both manufactured by Retina Implant AG (Reutlingen, Germany) which are taken as two datasets for evaluation of the clinical and laboratory reliability. The first one is Alpha IMS. Technical development, which consists mainly on modifications of the design and optimizing the control signals of the CMOS chip, led to the second generation, Alpha AMS. A schematic picture of the implant system is shown in Fig. 1. Both systems consist of a CMOS chip which is fixed on top of a foil substrate. In the two implant systems the CMOS chip has 1500 and 1600 photodiodes on an area of 3x3mm ^2^, respectively. The chip is placed subretinally in the eye. The photodiodes are collecting the light that falls through the natural pathway of the eye onto the chip. The amplified charge signal is then transferred by an electrode to the adjacent retinal layer. The retroauricularly placed subdermal coil that is enclosed in a hermetic ceramic housing is providing energy and control signals for the chip and is controlled by an external handheld device. The coil is connected by a power supply cable to the foil substrate. It consists of medical grade silicone encasing helically coiled gold wires. A return electrode is implanted close to the ceramic housing.

### Competing risk analysis of laboratory data

The whole system is divided into several components and all critical components are tested separately in laboratory environment, some of them under accelerated aging conditions at 60^∘^C to shorten test time (Carfagno and Gibson 1980; Hemmerich 1998). The tested components are: the CMOS chip, the foil substrate and the power supply cable. For these components lifetime is determined by laboratory experiments. For each tested component a specific lifetime distribution is determined from the laboratory data. As characteristic parameter for the distributions the value of the median life was used, which means that with a probability of 50 *%* the products will function for that period of time. The ceramic housing and the return electrode are expected to have a much longer component lifetime compared to the critical components. In the clinical study no failure due to ceramic housing or the return electrode was obeserved. Therefore they are neglected in the calculation of the reliability.

To determine the median lifetime of the CMOS chip several chips were tested under accelerated aging conditions at 60^∘^C. They were operated in phosphate buffered saline solution (PBS) using the normal handheld devices to guarantee the test conditions to be as close to normal operating conditions as possible. They are operated at gain and sensitivity levels that are close to the maximum levels that are typical for clinical use. The time-to-failure of each chip can be determined very precisely, because of the sudden drop of the continuously measured stimulation signal. A Weibull distribution (Wilker 2010) is fitted to the obtained device lifetimes and the median time is determined. According to the Arrhenius equation for chemical reaction rates the accelerated aging factor (AAF) between operating condition at *T*_*O*_ = 37^∘^C and test condition at *T*_*T*_ = 60^∘^C can be approximated by the 10-degree-rule (Hemmerich 1998)

1 $$ AAF= Q_{10}^{(T_{T}-T_{O} )/10}=2^{\Delta T/10} =4.92 $$

A reaction rate coefficient of *Q*_10_ = 2 is commonly assumed for aging of systems under moderate aging conditions and is typically applied for medical devices (Hemmerich 1998). We assumed a maximum runtime of 8 hours per day, which is based on information gathered from the patients during the clinical trial, because due to the observed failure mechanisms it is expected that the chip is only aging when it is in operation.

The lifetime of the polyimide foil substrate was tested by applying a constant voltage to all conducting traces of the substrate and measuring the leakage current from the foil substrate to a return electrode while the substrate is immersed in PBS. When the polyimide substrate starts to fail, a sudden increase of the leakage current by several decades can be observed. We again assume an accelerated aging factor of 4.92 but an operation time of 24h per day since we presume that the foil substrate is also aging when the implant is switched off. A Weibull distribution is used to determine the median life.

The power supply cable was tested by bending tests using an apparatus that simulates a periodic bending of a fixated power supply cable. The lifetime distribution of the power supply cable is approximated by an exponential distribution because all failures are assumed to be randomly distributed. The physical interpretation concerning our system is that sudden failures occur for example due to ruptures of the cables. As the number of eye movements largely depend on deflection angle from normal eye position it is hard to find a reliable value in literature for the typical number of eye movements per year. Considering the rather slow stimulation frequency of the implant, corresponding to a high eye fixation time, and the time the retinal implant is typically used, we estimate about 2 million eye movements per year (Rötting 2001; Takahashi and Atsumi 1997; Kokelmann and Wrobel 2012).

The lifetime of the whole system of our subretinal implant is estimated from these laboratory data by a competing risk analysis (NIST/SEMATECH 2013). It assumes that all components are connected in series and the whole system fails when one of the components fails. Therefore the reliability of the whole system *S*_*Sys*_ is the product of the reliabilities of all components.

2 $$ S_{Sys} (t)=S_{CMOS} (t)\cdot S_{Flex Substrate} (t)\cdot S_{Power Cable} (t)  $$

For each component the fraction of survived devices at time *t* is described by the reliability *S*(*t*) which is determined by fitting of the laboratory lifetime data. Depending on the type of distribution of the data points for each component either Weibull or Exponential distribution is chosen to determine the median life where *S*(*t*)=50 *%*. All component reliabilities are multiplied time-dependent. The resulting median life *τ* of the complete implant system can then be determined by

3 $$ S_{Sys}(\tau)=50~\%. $$

### Calculation of the clinical reliability

The multicenter trial (www.clinicaltrials.gov, NCT0102 4803) and its interim results are described in detail by Stingl et al. (2015). They showed, that with the retinal implant Alpha IMS a rudimentary vision could be restored in patients, that are blind from hereditary degenerations of photoreceptors. Patients were able to localise and recognize high-contrast objects. In this publication we focus on the clinical reliability of the two implants. Alpha IMS was implanted in 29 patients (in 2010-2013), but due to one reimplantation 30 implant systems are considered in the evaluation. Alpha AMS on the other hand was implanted in 10 patients during the multicenter trial (since 2014) and additionally in five patients as part of an ongoing clinical trial at NIHR Oxford (National Institute for Health Research, Oxford, GB, NCT02720640). For evaluation of the reliability both trials are analyzed in one dataset, since the device (Alpha AMS) is the same. One additional implantation of Alpha AMS that is not part of a clinical trial was also included for the calculation of the clinical reliability.

The functional life of the implants was defined by calculating the days between implantation and reported failure of the implant. The patients can clearly detect a malfunction of the implants by themselves when they no longer have any visual perception. Additional measurements using electroretinography equipment are carried out in the clinic to verify the reported failure of the device (Stingl et al. 2016). If a malfunction of the device is confirmed, the time of failure as reported by the patient is noted for the evaluation of the clinical reliability. In that way the lifetime of all implants in clinical use are continuously collected and evaluated.

The clinical reliability is estimated by two independent methods. One method is an actuarial analysis, resulting in a Kaplan-Meier-Plot. As the mean system lifetime for the Alpha AMS system cannot yet be predicted by the Kaplan-Meier method, a second method is used for estimating the expected mean lifetime using an exponential distribution.

#### Clinical reliability evaluated by Kaplan-Meier-Method

We evaluated the reliability of the implants by an actuarial analysis in compliance with ISO 5841-2:2014 with slight adaptation to our specific case. The devices were categorized depending on the status of the implant at each time step into the categories mentioned in Table 1.

**Table 1:** Device categorization for Kaplan-Meier-Method. Depending on the device function each implanted system is categorized at each time step

Category	
A	Device in service, no complication reported.
B	Device removed from service for reasons not
	related to the device functioning.
C	Device with malfunction confirmed.
D	Patient died, death unrelated to the
	functioning of the device.
L	Device is lost to follow-up. No complication
	or malfunction recorded.

In actuarial analysis the key variable to describe the reliability is the cumulative survival rate versus time. It can always be used when the distribution of the datapoints is not known in advance and a continuous evaluation is possible even when not all devices have failed. The Kaplan-Meier estimation is an empirical (non-parametric) procedure (Kaplan and Meier 1958). The cumulative survival corresponds to the estimated probability that a device is surviving for a specific period. It is a product of the survival fractions *P*(*t*), which describe the probability, that a device is surviving one specific time interval, calculated by

4 $$ P(t)=1-\frac{C(t)}{U(t)} $$

with the number of failed devices *C*(*t*) in that time interval and the units at risk *U*(*t*) which describe the number of devices that could have failed in that time step. The cumulative survival rate *S*(*t*) is calculated from the survival fraction *P*(*t*) by

5 $$ S(t)=\prod\limits_{t_{n}} P(t_{n} )=P(t)\cdot P(t-1)\cdot\ldots\cdot P(1) $$

Due to the small number of patients the confidence intervals, calculated by Greenwood’s formula (Kaplan and Meier 1958)

6 $$ \sigma^{2}(t)=S(t)^{2}\sum\limits_{t_{j}\le t}\frac{C(t_{j})}{U(t_{j})(U(t_{j})-C(t_{j}))} $$

are set to 68 *%* which is equivalent to one standard deviation *σ*(*t*).

#### Estimation of system lifetime from clinical data

The expected lifetime of the implants in the clinical trial was estimated assuming an exponential life distribution, also called HPP model (Homogeneous Poisson Process). It is a common method in reliability engineering to verify that a system meets the desired reliability requirements. Also, the clinical reliability of the system Alpha IMS shows a clearly exponential behavior. For that reason the same behavior is expected for the Alpha AMS system. In this model it is assumed, that the failure rate is constant. Due to this constant failure rate it is possible to estimate the characteristic lifetime of a system by adding all accumulated lifetimes of all measured devices and divide it by the number of failed devices. A one-sided lower confidence bound is calculated by dividing the accumulated lifetimes by a factor that depends on the number of occurred failures and the desired confidence level. It can be calculated from the Chi-Square distribution (NIST/SEMATECH 2013).

## Results

### Laboratory results of the components of Alpha IMS and Alpha AMS

A measured operational lifetime of the Alpha IMS CMOS chips of in average 723.5 h at 60^∘^C (Median) equals 3559.6 h at 37^∘^C for the assumed accelerated aging factor of 4.92. For an operation time of maximum 8 hours per day the median lifetime of the Alpha IMS CMOS chip can thus be estimated to 1.22 years.

The observed median lifetime of the Alpha IMS foil substrate was 226 days (60^∘^C). Again an acceleration factor of 4.92 was used. With this we expect a median lifetime of 3.05 years. To ensure the correctness of the applied acceleration factor additional tests at 37^∘^C were carried out, corresponding to real time aging. At this setting an acceleration factor of 4.8 could be determined.

For the power supply cable in average 3.14 million bending cycles could be performed until the cables failed. With expected 2 million eye movements per year this results in a median lifetime of 1.57 years.

The laboratory lifetime for the Alpha AMS components were determined correspondingly. All results for Alpha IMS and Alpha AMS components are summarized in Table 2. The system lifetime was then calculated as described in the methods section by a competing risk analysis. For Alpha IMS the median system lifetime is estimated to 0.7 years and for Alpha AMS 4.7 years.

**Table 2:** Median lifetime *τ* at which a given percentage *S*(*τ*) of Alpha IMS and Alpha AMS devices is still functioning. he total system lifetime is calculated by a product of the component reliabilities according to Eq. 2

Component	Alpha IMS	Alpha AMS
	*τ*[years]	*τ*[years]
CMOS chip	1.22 (50 *%*)	6.04 (50 *%*)
Foil substrate	3.05 (50 *%*)	12.7 (50 *%*)
Power supply cable	1.57 (50 *%*)	7.04 (50 *%*)
Total median system	0.7 years	4.7 years
lifetime *S*=50 *%*		

### Clinical results of the Alpha IMS and Alpha AMS clinical trials

**Fig. 2:**
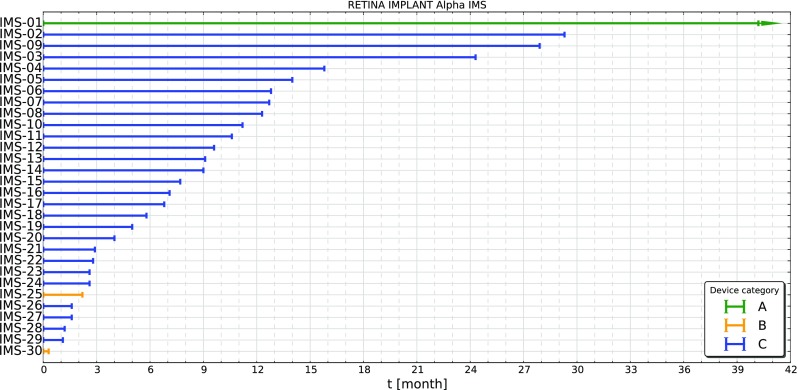
Duration of function of all implanted Alpha IMS systems. Almost all of them are reported as failed (Category C, *blue*), two are not functioning due to other reasons (Category B, *yellow*) and from one no malfunction was reported (Category A, *green*) from the patient

**Fig. 3:**
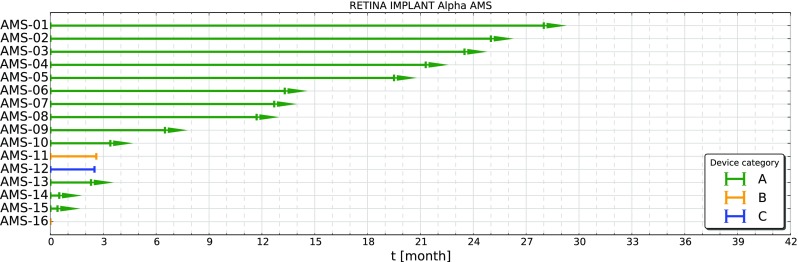
Duration of function of all implanted Alpha AMS systems. Except three systems all of them are still functioning (Category A, *green*), two failed due to procedural errors (Category B, *yellow*) and only one implant failed due to technical reasons (Category C, *blue*)

In Figs. 2 and 3 the duration of function of all implanted Alpha IMS and Alpha AMS systems up to now are shown. The colors refer to the categories described in Table 1. Arrow heads on the lines of category “A” devices (green) indicate, that these devices are still functioning and the measurement is ongoing. Almost all of the Alpha IMS implant systems are reported as failed, while for Alpha AMS most of the implants are still functioning. Three of the 16 implants are no longer working. However only one of these failures could be attributed to a malfunction of the device the other two devices were damaged possibly due to procedural errors. For that reason these two implants are considered as censored data in the evaluation of the Kaplan-Meier-Plot, because the malfunction is not related to the implant (ISO 5841-2:2014, category B, induced malfunction). However for the estimation of the mean device functionality time using an exponential model we included these devices as failures to include all possible failure mechanisms.

**Fig. 4:**
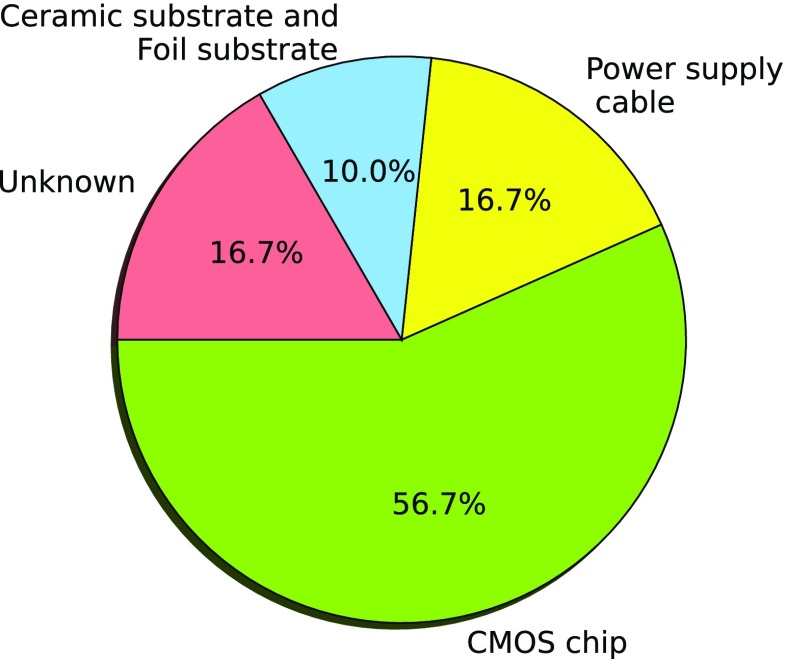
Failure mechanisms of explanted Alpha IMS implants. All explanted Alpha IMS systems were analyzed and degradations on implant components were used to determine the dominating failure mechanisms of each implant. The most critical component of the Alpha IMS system was corrosion of the CMOS chip

Most of the failed implants during the clinical trial were explanted to analyze the failure mechanisms. In Fig. 4 the relative percentage of all observed failure mechanisms of the explanted Alpha IMS devices are shown. It can be seen, that the most critical component of the implant system is the CMOS chip itself. In 57 *%* of the devices chip corrosion is observed.

### Clinical reliability of Alpha IMS and Alpha AMS

#### Kaplan-Meier-Method

Since the Alpha IMS subretinal implant was implanted already several years ago, the patients are no longer monitored. But, apart from one implant all of them are already reported as failed. With the clinical trial for Alpha AMS still ongoing the presented numbers are not final. Only data until June 2016 could be evaluated. The individual data are shown in Fig. 2 for Alpha IMS and in Fig. 3 for Alpha AMS.

**Fig. 5:**
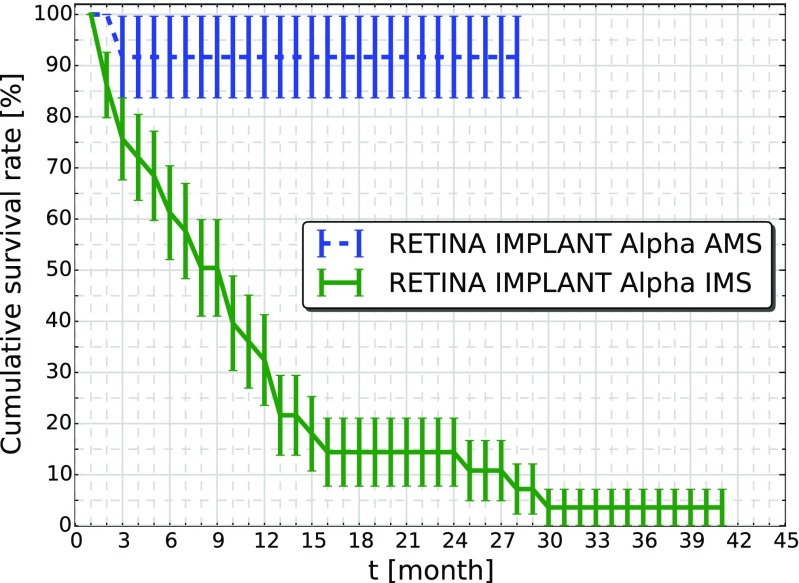
Kaplan-Meier-Plot of Alpha IMS and Alpha AMS devices. The cumulative survival rate describes the percentage of implant systems that functioned until month *t*. The error bars are calculated according to Greenwood’s formula. For the Alpha IMS system 50 *%* of the systems failed after 8 months, while for Alpha AMS only one technical device failure occurred until now. The time when 50 *%* of the Alpha AMS devices will be failed is not yet predictable

The cumulative survival of the Alpha IMS and the Alpha AMS is plotted in Fig. 5 as a Kaplan-Meier-Plot. The number of functional Alpha IMS devices decreased continuously during the first year after implantation according to an exponential behavior. The cumulative survival rate of 50 *%* (Median) was attained after 8 months mirroring the laboratory data where we estimated a lifetime of the total system of 0.7 years = 8.4 months.

For Alpha AMS only one device failure due to malfunctions of the system has occurred, thereby the survival rate of the devices is 92 *%* with a maximum implanted lifetime of 28 months. The median lifetime of 50 *%* survival is not predictable yet.

#### Lifetime estimation from clinical data

**Fig. 6:**
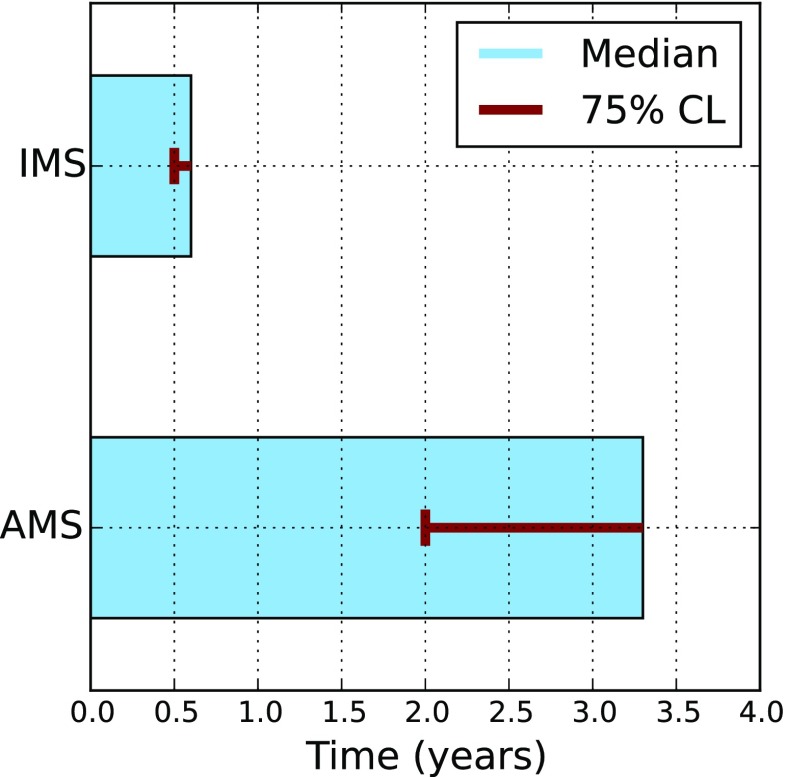
Median device functionality time of Alpha IMS and Alpha AMS with lower 75 *%* confidence level (CL) estimated from clinical data including technical and procedural related failures. For Alpha IMS the median device functionality time is estimated to be 0.6 years and a lower 75 *%* confidence bound of 0.5 years and for Alpha AMS the median lifetime is expected to be 3.3 years with a lower 75 *%* confidence bound of 2.0 years

As described in the methods an exponential model is used to estimate the system lifetime of the implants from clinical data. For Alpha IMS the total runtime of 30 devices is 23 years with 29 failures resulting in a median lifetime of 0.6 years. The lower confidence bound for 75 *%* is 0.5 years. For Alpha AMS and 16 patients a total runtime of 14.5 years was accumulated. Two of the implants failed after procedural errors and one implant failed due to technical reasons, resulting in an estimated median lifetime of 3.3 years and a lower confidence bound for 75 *%* of 2.0 years. This suggests an increase in lifetime by at least a factor of 4. These results are depicted in Fig. 6.

## Discussion

Up to now only three retinal devices have a market approval. Several other approaches are under development and no long-term reliability data are available (Ayton et al. 2014; Guenther et al. 2012; Luo and da Cruz 2014; Menzel-Severing et al. 2012; Yue et al. 2016). The other commercially available retinal prostheses use an external camera in combination with an epiretinally placed electrode unit (Ho et al. 2015). In that case it is possible to encapsulate all implanted electronics in a hermetic housing, reducing the parts that are in direct contact with body fluids to passive stimulation electrodes. Thereby a long device lifetime can be reached (Ho et al. 2015). However, although the visual field can be larger for the epiretinal approach, the drawback of such a configuration is that it cannot be easily scaled up to a large number of electrodes, subsequently limiting the achievable visual acuity. Our approach of a subretinal implant already now allows a large number of stimulation electrodes and it has been demonstrated, that a Snellen visual acuity of 20/546 is possible (Stingl et al. 2013). Additionally it has the advantage that the natural eye movements can be utilized. Considering the lack of alternative treatments for people who are blind due to retinitis pigmentosa a device with a long lifetime is crucial.

However, the first generation of the subretinal implant, the Alpha IMS, did not meet this criterion. In the patient the Alpha IMS lasted less than a year (median lifetime 0.6 years with a lower 75 *%* confidence bound of 0.5 years). Since the visual results of the Alpha IMS implant itself were promising (Stingl et al. 2015), technical developments led to the second subretinal implant Alpha AMS. The clinical trial is not yet finished, but the clinical data of the Alpha AMS system that are available up to now suggest a median system lifetime of 3.3 years with a lower 75 *%* confidence bound of 2.0 years. These results mean that the lifetime of the implants could be increased at least by a factor of 4.0.

The clinical results are in good agreement with our laboratory results, which are based on accelerated aging tests and a competing risk analysis. The laboratory result of the estimated system lifetime is slightly higher because they include only technical failures, while in the calculation of the device lifetime from clinical data also failures due to procedural errors are included. The laboratory results suggest a total median lifetime of the Alpha IMS system of 0.70 years. This shows that our laboratory experiments can give an acceptable estimation of the actual clinical reliability. Therefore we expect that we can also infer from the laboratory experiments from Alpha AMS to the expected clinical reliability. Also the failure mechanisms that are observed in the clinical trial at Alpha IMS systems match the failure mechanisms determined in laboratory tests which shows the suitability of the laboratory model. In laboratory experiments the CMOS chip showed the shortest lifetime (see Table 2), and in the clinical trial the CMOS chip was the dominating failure mechanism (Fig. 4). Also for Alpha AMS the results of laboratory tests, which show an expected median lifetime of 4.7 years, are in good agreement with the clinical data that are available up to now. Hence, laboratory tests and results from the ongoing clinical trial show that a lifespan of several years is now possible with the Alpha AMS system. Taking into account the possible option of reimplantation, which is rather uncomplicated since the implant is only held in place by the inner eye pressure, the Alpha AMS is a considerable option for blind RP patients. Further increase of the lifetime of the implant might be possible through the implementation of sophisticated coating materials as additional passivation layers, that can better withstand the harsh conditions. Currently several possibilities are under developement, like ultrananocrystalline diamond (Xiao et al. 2006), SiC coatings (Lei et al. 2016) or encapsulation of the electronics in parylene C (Chang et al. 2013). In addition with further opimizations of the design to avoid corrosion and a possible reduction of control voltages another considerable increase in the lifetime might be possible.

## Conclusion

In this paper we showed reliability estimations for the subretinal implants Alpha IMS and Alpha AMS (Retina Implant AG, Reutlingen). The clinical reliability was determined by actuarial analysis and using an exponential distribution to estimate the expected system lifetime. Laboratory data of implant components were combined in a competing risk analysis. From clinical, as well as from laboratory data we can estimate a device functionality of several years of the Alpha AMS system which is a significant increase compared to the previous product generation Alpha IMS.
